# Predictive value of Caprini risk assessment model, D-dimer, and fibrinogen levels on lower extremity deep vein thrombosis in patients with spontaneous intracerebral hemorrhage

**DOI:** 10.3389/fneur.2024.1370029

**Published:** 2024-05-30

**Authors:** Xia Fang, Yi Shen, Mei Wang, Lihong Dai, Linlin Shi, Feng Zhang, Congcong Chen, Yuan Yuan

**Affiliations:** ^1^Department of Nursing, Shanghai General Hospital, Shanghai Jiao Tong University School of Medicine, Shanghai, China; ^2^Department of Nursing, Xinqiao Town Community Health Service Center, Songjiang District, Shanghai, China

**Keywords:** Caprini risk assessment model, spontaneous intracerebral hemorrhage, D-dimer, fibrinogen, lower extremity deep vein thrombosis

## Abstract

**Introduction:**

Research indicates that individuals experiencing hemorrhagic stroke face a greater likelihood of developing lower extremity deep vein thrombosis (DVT) compared to those with ischemic stroke. This study aimed to assess the predictive capacity of the Caprini risk assessment model (RAM), D-dimer (D-D) levels, and fibrinogen (FIB) levels for lower extremity DVT in patients with spontaneous intracerebral hemorrhage (sICH).

**Methodology:**

This study involved a retrospective analysis of medical records from all sICH patients admitted to Shanghai General Hospital between June 2020 and June 2023. Within 48 h of admission, patients underwent routine screening via color Doppler ultrasonography (CDUS). Patients were categorized into the DVT and control groups based on the occurrence of lower extremity DVT during hospitalization. Differences in Caprini RAM, D-dimer, and FIB levels between the two groups were compared. The sensitivity and specificity of combined Caprini RAM, peripheral blood D-dimer, and FIB levels in predicting lower extremity DVT in sICH patients were analyzed. Receiver operating characteristic (ROC) curves assessed the overall predictive accuracy of Caprini RAM, D-D, and FIB levels.

**Results:**

The study involving 842 sICH patients revealed 225 patients with DVT and 617 patients without DVT. Caprini RAM, D-D, and FIB levels were significantly higher in the DVT group compared to the control group (*P* < 0.05). Sensitivity values for Caprini RAM, D-D, and FIB levels in predicting lower extremity DVT in sICH patients were 0.920, 0.893, and 0.680, respectively, while specificities were 0.840, 0.680, and 0.747, respectively. The ROC curve analysis demonstrated an area under the curve (AUC) of 0.947 for combined DVT prediction, with 97.33% sensitivity and 92.00% specificity, indicating superior predictive value compared to individual applications of Caprini RAM, D-D, and FIB levels.

**Conclusion:**

The combined utilization of Caprini RAM, D-D, and FIB levels holds significant clinical relevance in predicting lower extremity DVT in sICH patients.

## Introduction

Spontaneous Intracerebral hemorrhage (sICH) is a severe subtype of stroke with high mortality and disability rates, and sICH accounts for approximately 10%−15% of all strokes (1). Deep vein thrombosis (DVT) is common in stroke patients due to hemiplegia, long-term bed rest, and other reasons. Depending on literature reports (2–4), the incidence of DVT in acute stroke patients is 45%, and the incidence of DVT in severe hemiplegia patients is as high as 60%~75%. DVT prophylaxis might be a potential target to improve clinical outcomes after sICH. In addition, the optimal approach for DVT prophylaxis in an sICH patient is a challenge of balancing the reduction in the incidence of DVT without risking an increase in sICH. However, no reliable scoring system is currently available to predict in-hospital DVT after sICH in routine clinical practice or clinical trials (5).

Plasma D-D level is a valuable diagnostic test for the exclusion of DVT, but it is uncertain whether plasma D-D level can predict the occurrence of DVT in patients with sICH. Ogata et al. (6) and Cheng et al. (7) found that high plasma D-D level was an independent risk factor for DVT in patients with sICH. Chen et al. (8) suggested that D-D combined with albumin could well predict DVT in patients with sICH. However, Johansson et al. (9) found that high plasma D-D concentrations were associated with an increased risk of future cerebral hemorrhage. Therefore, one of the aims of this study was to investigate whether plasma D-D levels at admission were associated with the development of DVT in patients with sICH.

Studies Wolberg and Sang (10) and Aleman et al. (11) have shown the mechanism by which fibrinogen (FIB) levels affect the size, composition, and stability of venous thrombosis. However, Chitsaz et al. (12) found that there was no significant difference in average FIB level between sICH and ischemic stroke patients. This study further explores the influence of FIB level on DVT in patients with sICH. The Caprini risk assessment model (RAM) has been extensively used to predict venous thromboembolism (VTE) in clinical diagnosis and treatment (13). However, the Caprini RAM lacks specificity in predicting DVT. The purpose of this study was to investigate the clinical value of Caprini RAM, D-D, and FIB levels in predicting the occurrence of lower extremity DVT in patients with sICH.

## Subjects and methods

### Study population

**Figure 1:**
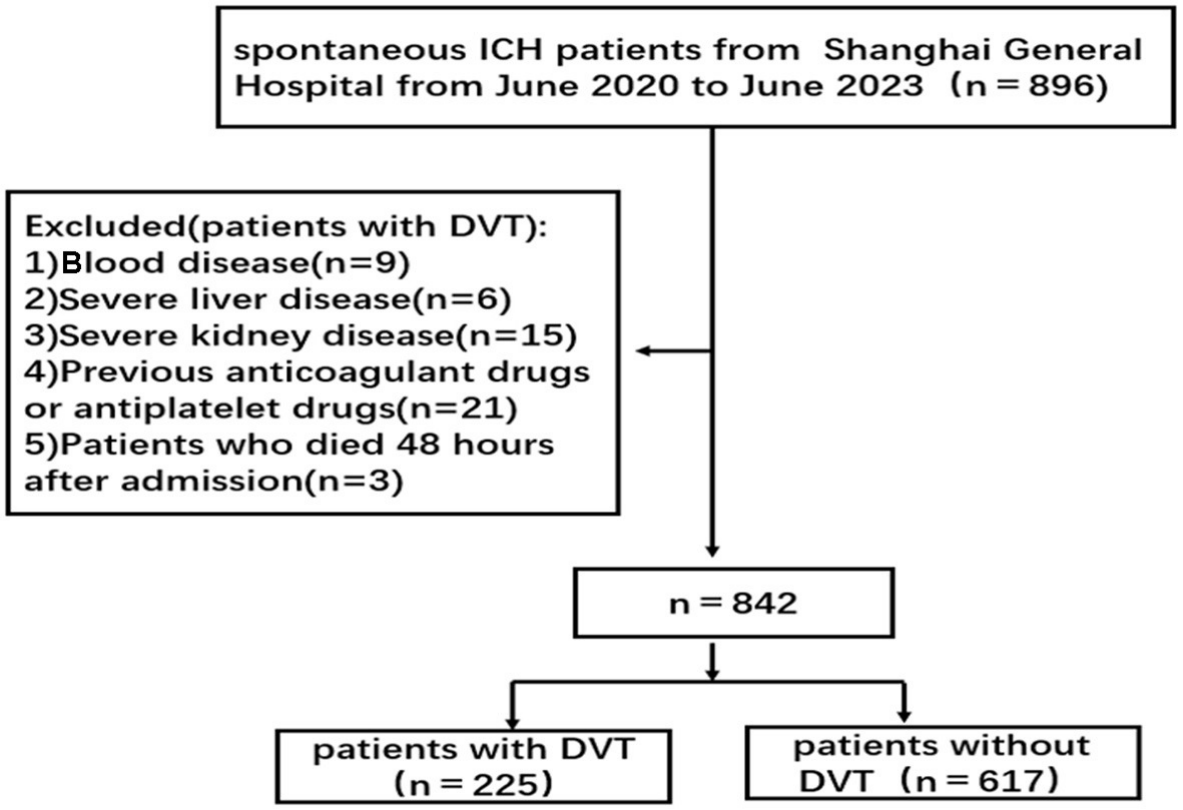
The flow of patient selection. ICH, intracerebral hemorrhage; DVT, deep venous thrombosis.

This research involved consecutive sICH patients from the emergency internal medicine of the Shanghai General Hospital from June 2020 to June 2023 (Figure 1). The patients were divided into the DVT group and the control group according to whether lower extremity DVT occurred. Patients who fulfilled each of the inclusion criteria were included: patients with age ≥ 18 years old; patients who met the diagnostic criteria for sICH (14); patients who had first onset and for an acute period (duration < 14 days); and patients with no lower extremity DVT at admission.

The following were the exclusion criteria: patients with severe coagulation dysfunction and blood disease; patients with severe liver and kidney diseases; patients who used anticoagulant drugs or antiplatelet drugs 3 months before admission; patients with incomplete electronic data; and patients who died 48 h after admission.

This study was approved by the ethics committee of the Shanghai General Hospital [2023186]. Each procedure adhered to the Helsinki Declaration. Due to the application of a retrospective study design, written consent was not required.

#### Ultrasonic examination method and standard for diagnosing DVT

Lower extremity Doppler examination was performed again during hospitalization when the patient had symptoms of deep venous thrombosis. We used Vivid 7 Dimension (GE, USA) with the 7–10-Hz linear probe. The patient was in a supine position with legs apart, slightly external rotation, and abduction. The following areas of the leg were examined: the iliac veins and calf veins, the superficial femoral vein, the common femoral vein at the inguinal ligament, and the popliteal vein at the knee joint line traced down to the point of the trifurcation of the calf veins. Veins were scanned in the transverse plane only. Diagnostic criteria for deep venous thrombosis included echoes in the venous cavity of varying intensity, plaque partially occupying the lumen of the blood vessel; if the probe was pressurized, the venous lumen could not be completely crushed. When the deep vein was partially embolized, the blood flow signal detected by the probe showed the blood flow filled with defects; only after squeezing the distal limb could a small amount of blood flow be seen. In contrast, when the deep vein was completely embolized, the probe could not detect blood flow at the lesion, and the distal blood flow did not increase when squeezed.

#### 2013 Caprini RAM

**Figure 2:**
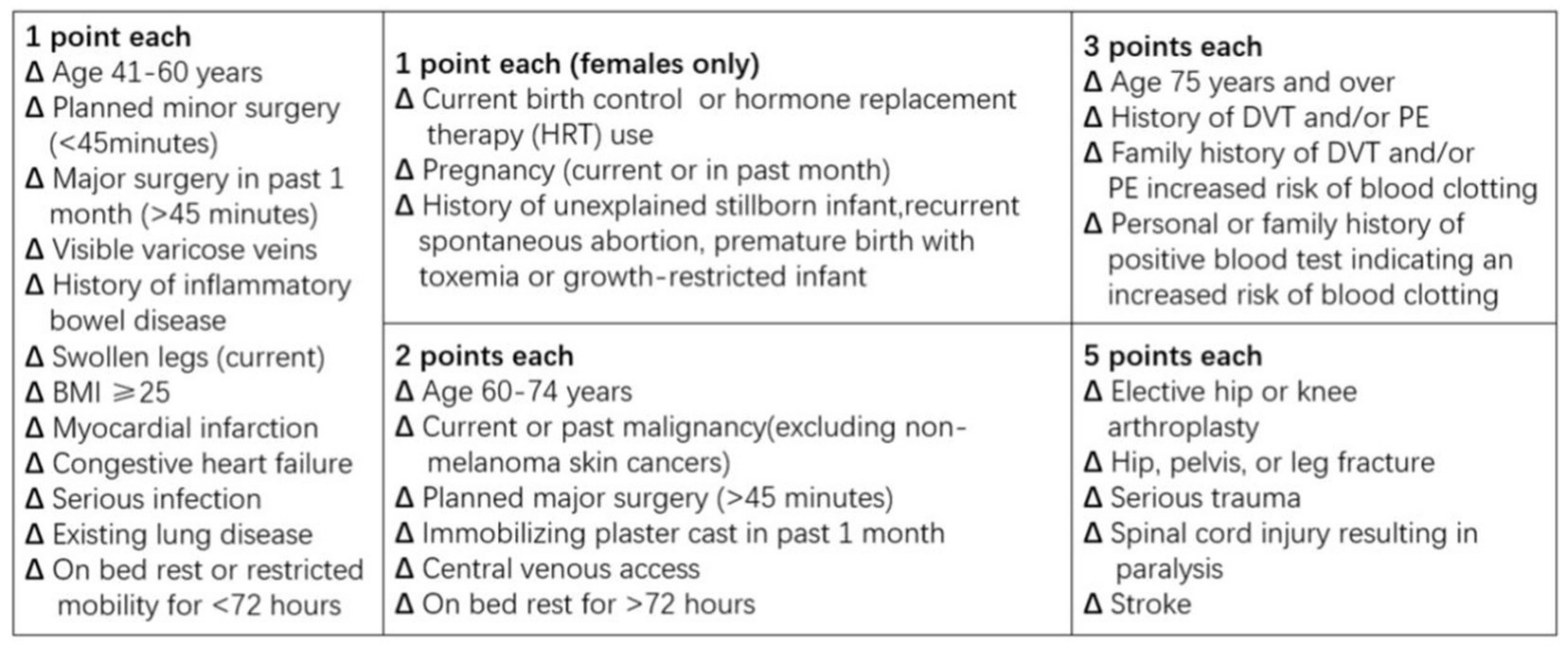
Caprini risk assessment model 2013.

In this study, we applied the Caprini RAM edition 2013 (Figure 2), which excluded some expensive and impractical laboratory variables and optimized some risk factors, unlike the previous version. In Figure 2, each point value corresponds to a specific risk factor, varying from 1 to 5. The sum of these scores is linked to an individual's risk of developing lower extremity DVT. Because all patients in this study were sICH patients, all Caprini RAM values were >5.

### Data collection

All enrolled patients were analyzed with our hospital's electronic medical records. The general information includes sex, age, hypertension, diabetes, surgical intervention, smoking history, history of alcohol, pulmonary infection, platelet count, activated partial thromboplastin time (APTT), and prothrombin time (PT). All patients received basic prophylaxis of thrombus (including elevating the affected limb, joint movement, promoting lower limb muscle contraction, and deep breathing, while quitting smoking and alcohol), and the number of patients who received mechanical prophylaxis with elastic stockings or intermittent pneumatic compression on admission was recorded. The time from sICH onset to the diagnosis of lower extremity DVT and the time from sICH onset to anticoagulation initiation was recorded. Whether any of the sICH patients with lower extremity DVT received anticoagulant therapy was decided by the clinicians according to the patient's condition, and the preference of the patients' proxy was not influenced by the researchers. Fasting venous blood D-D and FIB levels were collected within 48 h after admission. The D-D instrument used was a mini VIDAS automatic immunofluorescence analyzer (France Biomeriette), and the double-antibody sandwich immunofluorescence method was used for detection. The FIB instrument used was a Sysmex CS-5100 automatic coagulation instrument [Sysmex Medical Electronics (Shanghai) Co., Ltd.], and the detection method used was scattered light turbidimetry. In the emergency medical ward of our hospital, all patients were routinely screened by using color Doppler ultrasonography (CDUS) within 48 h of admission. The Caprini RAM is scored by two senior nurses within 48 h of admission using the Caprini scoring form. The investigators were trained to ensure the accuracy of the data.

### Statistical analysis

Statistical analyses were performed with SPSS (version 22.0). Continuous variables were expressed as mean and standard deviation (SD) or median and interquartile range (IQR). In univariate analysis, the Chi-square or Fisher's exact test was used to compare categorical variables between groups, and the Mann–Whitney test or independent *t*-test was employed to compare continuous variables between groups. The predictive value of Caprini score, D-dimer, and fibrinogen levels for DVT in patients with sICH was evaluated by calculating the area under the receiver operating characteristic curve. Meanwhile, specificity, sensitivity, rate of missed diagnosis, and misdiagnosis rate were calculated at the maximum Youden index. All tests were two-tailed, and *P* < 0.05 was considered statistically significant.

## Results

### Comparison of clinical baseline data between the two groups

A total of 842 eligible patients with sICH were included in this study (Figure 1). The incidence of lower extremity DVT in this study was 26.72%. There was no significant difference in sex composition, age, hypertension, diabetes, surgical intervention, smoking history, history of alcohol, pulmonary infection, platelet count, APTT, and PT between the two groups (*P* > 0.05) (Table 1).

**Table 1:** A comparison of general data between the DVT group and the control group.

**Variables**	**Control group (*n =* 225)**	**DVT group (*n =* 617)**	**χ^2^*/t***	***P*-value**
**Sex**				
Male [*n*(%)]	147 (65.33)	361 (58.51)	3.208	0.073
Female [*n*(%)]	78 (34.67)	256 (41.49)		
Age (x̄ **±s**)	60.57 ± 8.78	61.21 ± 8.41	−0.996	0.321
Hypertension [*n*(%)]	162 (72.00)	485 (78.61)	0.539	0.463
Diabetes mellitus [*n*(%)]	38 (16.89)	93 (15.07)	0.300	0.583
Surgical intervention, *n* (%)	46 (20.44)	170 (27.55)	2.656	0.103
Smoking history [n(%)]	81 (36.00)	175 (28. 36)	2.363	0.124
History of alcohol [n(%)]	63 (28. 00)	134 (21.71)	2.203	0.137
Pulmonary infection [n(%)]	66 (29.33)	200 (32.41)	0.381	0.537
Platelet count/(10^9^/L)	214.80 ± 58.51	218.62 ± 64.56	0.481	0.643
APTT	34.06 ± 4.64	34.52 ± 9.46	0.836	0.427
PT	13.09 ± 0.83	13.21 ± 1.23	1.479	0.177

APTT, activated partial thromboplastin time; PT, prothrombin time.

### A comparison of Caprini RAM between the DVT group and the control group

**Figure 3:**
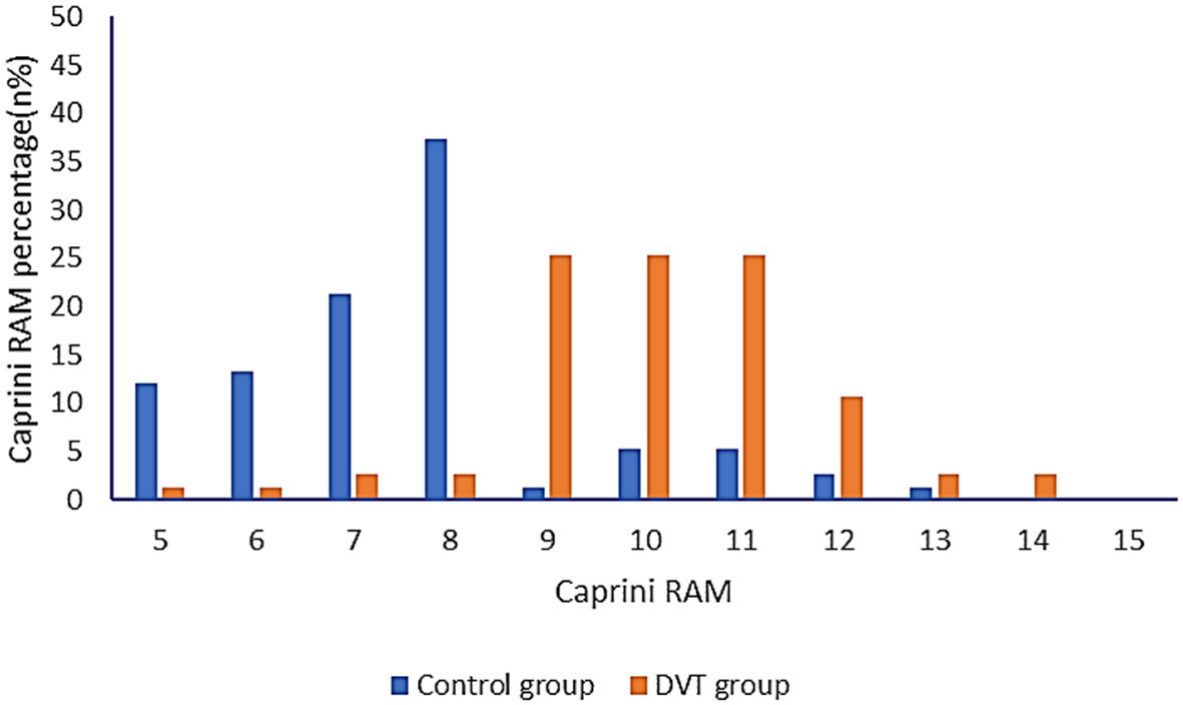
A comparison of Caprini RAM between the control group and the DVT group.

The Caprini RAM of the DVT group and the control group were compared. In total, 93.33% of patients had a score of ≥9 in the DVT group and 82.67% in the control group had a score of < 9. According to the ROC curve, when the critical value is 8.26, the Youden index reaches the highest level, and the corresponding sensitivity is 92%, and the specificity is 84% (Figure 3, Table 3). Therefore, when the Caprini RAM score is ≥5 and < 8.26, patients are at low risk of lower extremity DVT; when the Caprini RAM score is >8.26, patients are at high risk of lower extremity DVT.

### A comparison of Caprini RAM, D-D, and FIB levels between the two groups

The Caprini RAM, D-D, and FIB levels in the DVT group were higher than those in the control group, and the differences were statistically significant (*P* < 0.05) (Table 2).

**Table 2:** A comparison of Caprini RAM, D-D, and FIB levels between the two groups (x̄ **±s**).

**Group**	**Number**	**Caprini RAM**	**FIB (g/L)**	**D-D (mg/L)**
Control group	617	7.61 ± 1.77	3.59± 1.06	4.10 (2.20, 7.00)
DVT group	225	10.15 ± 1.57	4.51± 1.35	8.23 (4.50, 16.03)
*t/Z*		9.196	−4.629	−6.927
*P*-value		< 0.001	< 0.001	< 0.001

### Predictive value of Caprini RAM, D-D, and FIB levels for lower extremity DVT

**Figure 4:**
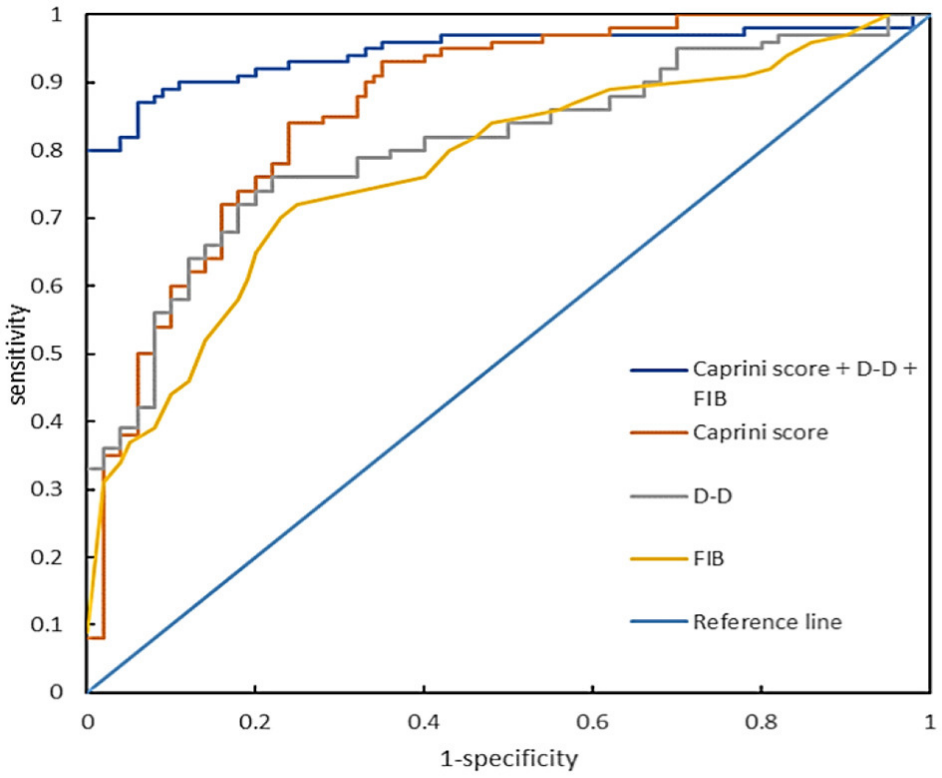
Receiver operating characteristic curve of Caprini RAM, D-D, and FIB levels alone or in combination during hospitalization for predicting lower extremity DVT complications in patients with sICH.

By plotting the receiver operating characteristic (ROC) curve, we obtained the diagnostic indicators corresponding to the Caprini RAM, the optimal diagnostic critical values of D-D and FIB, and the sensitivity, specificity, rate of missed diagnosis, misdiagnosis rate, and AUC (Area under curve). The sensitivity of the Caprini RAM, D-D, and FIB combined to predict lower extremity DVT in sICH patients was 97.33%, specificity was 92%, missed diagnosis rate was 2.67%, misdiagnosis rate was 8.00%, and the AUC was 0.947 (*P* < 0.05) (Figure 4, Table 3).

**Table 3:** Diagnostic effect of single predictor and combined predictor.

**Index**	***Cut-off***	**Sensitivity (%)**	**Specificity (%)**	**Rate of missed diagnosis (%)**	**Misdiagnosis rate (%)**	**AUC**
Caprini RAM	8.26	92.00	84.00	8.00	16.00	0.869
FIB	3.9 g/L	68.00	74.67	32.00	25.33	0.702
D-D	5.625 mg/L	89.33	68.00	10.67	32.00	0.817
Caprini RAM + D-D + FIB		97.33	92.00	2.67	8.00	0.947

### Anticoagulation status

Of the 842 patients with sICH, no patients received preventive anticoagulant therapy. The time from sICH to the diagnosis of lower extremity DVT was 10.5 (7.23–22) days, respectively. A total of 56.9% (128/225) of the sICH patients with VTE were administered anticoagulant therapy, including subcutaneous low molecular weight heparin of 4,000 units once daily, rivaroxaban tablets of 15 mg or 20 mg once daily, and warfarin adjusted according to an international normalized ratio between 2 and 3. Hemorrhage after anticoagulation occurred in 11 patients (8.6%), including 4 cases of gastrointestinal bleeding, 3 cases of hematuria, 2 cases of recurrent cerebral hemorrhage, and 2 cases of hemoptysis. One patient taking warfarin experienced severe gastrointestinal bleeding, and the other reported events were all non-severe.

## Discussion

DVT is the most common complication of sICH. Treatment options for cerebral hemorrhage and DVT are contradictory. The treatment of sICH focuses on hemostasis to avoid further worsening of cerebral hemorrhage, while the prevention and treatment of DVT are mainly anticoagulation, which has the risk of bleeding. Therefore, in order to reduce the further worsening of sICH and prevent thrombosis in patients with sICH complicated by lower extremity DVT, it is inevitable for clinicians to formulate appropriate treatment strategies (15–18).

The Caprini RAM (19–21) is one of the most commonly used individualized VTE risk assessment scales. However, as a diagnostic tool for DVT alone, the Caprini RAM still has some shortcomings, and the diagnostic results are not accurate enough. The heterogeneity of the Caprini RAM limits its practicality (22). It has been reported that when the threshold CRS is 7–11, the risk of VTE in the patients increases significantly (23). In this study, based on the original Caprini RAM, patients with sICH who scored in the “highest risk” category (score ≥ 5) were further substratified. This substratification in the “highest risk” category showed the risk of developing lower extremity DVT events was significantly higher among patients with a Caprini score of >8.26, compared to those with a Caprini score of 5–8. According to the ROC curve, it was found that when the cut-off value was 8.26, the Youden index reached the highest level, and its corresponding sensitivity was 92% and specificity was 84%. Therefore, we suggest that Caprini RAM can be used as one of the predictors of DVT in patients with sICH. When the score is ≥9, the patients are at high risk; when the score is < 9, the patients are at low risk of developing lower extremity DVT. Since this study is a single-center retrospective study, further large sample and prospective studies are needed.

D-D originates from fibrinolytic cross-linked fibrin degradation induced by plasminase. As a soluble fibrin degradation product, D-D is an important biomarker for secondary fibrinolysis and coagulation activation and has important reference value for eliminating venous thromboembolism (VTE), thus evaluating VTE recurrence and determining the optimal duration of anticoagulation therapy (24–26). However, its prediction of whether a patient will develop DVT in the future is uncertain. Studies Chopard et al. (27) have shown that D-D has a negative predictive value of 92% in the normal range, thus D-D alone cannot be used to eliminate lower extremity DVT. This study showed that the D-D level in the DVT group was significantly higher than that in the control group, with a sensitivity of 89.33% and a specificity of 68.00%, and the AUC was 0.817 (*P* < 0.05). These results indicate that the increase of D-dimer level can predict the formation of DVT in patients with sICH.

FIB is the highest concentration of coagulation factor in plasma, also known as coagulation factor I. The increase of FIB level in blood can promote coagulation dysfunction, shorten coagulation time, increase the risk of thrombosis, and promote the occurrence of lower extremity DVT (28–30). This study showed that the level of FIB in the DVT group was higher than that in the control group. Its sensitivity was 68.00%, specificity was 74.67%, and the AUC was 0.702. The results indicated that the increase in fibrinogen level was important in predicting DVT in patients with sICH. However, FIB could be increased under the conditions of tumor, chronic inflammation, diabetes, stress reaction, etc. FIB has the disadvantages of poor specificity and a high false positive rate in the diagnosis of lower extremity DVT when applied alone (31, 32).

To the best of our knowledge, this is the first study to combine Caprini RAM, D-D, and FIB levels to predict lower extremity DVT in patients with sICH. The results suggest that the combined application of Caprini RAM, D-D, and FIB levels in predicting the occurrence of lower extremity DVT in patients with sICH has a high sensitivity of 97.33%, a specificity of 92.00%, a low rate of missed diagnosis of 2.67%, misdiagnosis of 8.00%, and an AUC of 0.947. Wang et al. (33) plotted the ROC curve of white blood cells as predictors of lower extremity DVT, with an AUC of 0.722 (sensitivity of 87.10% and specificity of 61.29%). Chen et al. (8) plotted the ROC curve of the combination of D-D and albumin as predictors of lower extremity DVT, with an AUC of 0.759. The combination of Caprini RAM, D-D, and FIB levels is of great significance for predicting the occurrence of lower extremity DVT in patients with sICH, and the combined application of the three can improve the prediction efficiency.

This trial has several limitations. The first limitation is that this is a retrospective study. In this study, patient information was collected through electronic medical records and nursing records. A limitation of retrospective investigation studies is the risk of recall bias. This can impact the credibility and accuracy of the study's results. The second limitation is only patients with sICH in one hospital were selected, and the results of this study may be biased due to the small sample size. The third limitation is after the patient's color ultrasound is negative at the first admission, the second ultrasound will be performed when the patient has DVT symptoms, and the second color ultrasound will not be performed for patients with no symptoms, which will cause certain missed diagnoses. Therefore, this bias is unavoidable, and its impact on the results of the study is uncertain.

## Conclusion

The combined application of Caprini RAM, D-D, and FIB levels has important clinical value in predicting lower extremity DVT in patients with sICH.

## Data availability statement

The original contributions presented in the study are included in the article/supplementary material, further inquiries can be directed to the corresponding author.

## Ethics statement

The studies involving humans were approved by the Medical Ethics Committee of Shanghai General Hospital. The studies were conducted in accordance with the local legislation and institutional requirements. The participants provided their written informed consent to participate in this study. Written informed consent was obtained from the individual(s) for the publication of any potentially identifiable images or data included in this article.

## Author contributions

XF: Data curation, Methodology, Writing – original draft, Writing – review & editing. YS: Data curation, Investigation, Methodology, Supervision, Validation, Writing – review & editing. MW: Data curation, Investigation, Methodology, Project administration, Resources, Supervision, Writing – review & editing. LD: Formal analysis, Investigation, Methodology, Project administration, Resources, Supervision, Validation, Writing – review & editing. LS: Data curation, Investigation, Methodology, Project administration, Supervision, Validation, Writing – original draft. FZ: Data curation, Investigation, Methodology, Project administration, Resources, Validation, Writing – original draft. CC: Data curation, Investigation, Methodology, Project administration, Resources, Supervision, Validation, Writing – review & editing. YY: Data curation, Methodology, Project administration, Resources, Supervision, Validation, Writing – original draft, Writing – review & editing.

## References

[1] Sheth KN. Spontaneous intracerebral hemorrhage. N Engl J Med. (2022) 387:1589–96. 10.1056/NEJMra2201449 36300975

[2] Tøndel BG Morelli VM Hansen JB Braekkan SK. Risk factors and predictors for venous thromboembolism in people with ischemic stroke: a systematic review. J Thromb Haemost. (2022) 20:2173–86. 10.1111/jth.15813 35815351 PMC9796787

[3] Liu LP Zheng HG Wang DZ Wang YL Hussain M Sun HX. Risk assessment of deep vein thrombosis after acute stroke: a prospective study using clinical factors. CNS Neurosci Ther. (2014) 20:403–10. 10.1111/cns.12227 24612485 PMC6493054

[4] Mazzolai L Ageno W Alatri A Bauersachs R Becattini C Brodmann M. Second consensus document on diagnosis and management of acute deep vein thrombosis: updated document elaborated by the ESC Working Group on aorta and peripheral vascular diseases and the ESC Working Group on pulmonary circulation and right ventricular function. Eur J Prev Cardiol. (2022) 29:1248–63. 10.1093/eurjpc/zwab088 34254133

[5] Ji R Wang L Liu X Wang D Wang W Zhang R. A novel risk score to predict deep vein thrombosis after spontaneous intracerebral hemorrhage. Front Neurol. (2022) 13:930500. 10.3389/fneur.2022.930500 36388194 PMC9650187

[6] Ogata T Yasaka M Wakugawa Y Inoue T Ibayashi S Okada Y. Deep venous thrombosis after acute intracerebral hemorrhage. J Neurol Sci. (2008) 272:83–6. 10.1016/j.jns.2008.04.032 18555486

[7] Cheng X Zhang L Xie NC Ma YQ Lian YJ. High plasma levels of D-dimer are independently associated with a heightened risk of deep vein thrombosis in patients with intracerebral hemorrhage. Mol Neurobiol. (2016) 53:5671–8. 10.1007/s12035-015-9487-5 26491025

[8] Chen L Zhang W Liu C. Combination of D-dimer and albumin in the prediction of deep vein thrombosis in patients with spontaneous intracerebral hemorrhage. Clin Appl Thromb Hemost. (2023) 29:10760296231165054. 10.1177/10760296231165054 36946100 PMC10034306

[9] Johansson K Jansson JH Johansson L Wiklund PG Nilsson TK Lind M. D-Dimer is associated with first-ever intracerebral hemorrhage. Stroke. (2018) 49:2034–9. 10.1161/STROKEAHA.118.021751 30354971

[10] Wolberg AS Sang Y. Fibrinogen and factor XIII in venous thrombosis and thrombus stability. Arterioscler Thromb Vasc Biol. (2022) 42:931–41. 10.1161/ATVBAHA.122.317164 35652333 PMC9339521

[11] Aleman MM Walton BL Byrnes JR Wolberg AS. Fibrinogen and red blood cells in venous thrombosis. Thromb Res. (2014) 133:S38–S40. 10.1016/j.thromres.2014.03.017 24759140 PMC4003903

[12] Chitsaz A Mousavi SA Yousef Y Mostafa V. Comparison of changes in serum fibrinogen level in primary intracranial hemorrhage (ICH) and ischemic stroke. ARYA Atheroscler. (2012) 7:142–145. 23205046 PMC3448377

[13] Lobastov K Urbanek T Stepanov E Lal BK Marangoni J Krauss ES. The thresholds of Caprini score associated with increased risk of venous thromboembolism across different specialties: a systematic review. Ann Surg. (2023) 277:929–37. 10.1097/SLA.0000000000005843 36912040

[14] Greenberg SM Ziai WC Cordonnier C Dowlatshahi D Francis B Goldstein JN. 2022 Guideline for the management of patients with spontaneous intracerebral hemorrhage: a guideline from the American Heart Association/American Stroke Association. Stroke. (2022) 53:e282–361. 10.1161/STR.0000000000000407 35579034

[15] Yamashita Y Morimoto T Muraoka N Oyakawa T Umetsu M Akamatsu D. Edoxaban for 12 months versus 3 months in patients with cancer with isolated distal deep vein thrombosis (ONCO DVT Study): an open-label, multicenter, randomized clinical trial. Circulation. (2023) 148:1665–76. 10.1161/CIRCULATIONAHA.123.066360 37638968

[16] CLEAR III Trial Investigators. Venous thromboembolism after intraventricular hemorrhage: results from the CLEAR III trial. Neurosurgery. (2019) 84:709–16. 10.1093/neuros/nyy189 29788198 PMC8133352

[17] Ding D Sekar P Moomaw CJ Comeau ME James ML Testai F. Venous thromboembolism in patients with spontaneous intracerebral hemorrhage: a multicenter study. Neurosurgery. (2019) 84:E304–10. 10.1093/neuros/nyy333 30011018 PMC12311975

[18] Sprügel MI Sembill JA Kuramatsu JB Gerner ST Hagen M Roeder SS. Heparin for prophylaxis of venous thromboembolism in intracerebral haemorrhage. J Neurol Neurosurg Psychiatry. (2019) 90:783–91. 10.1136/jnnp-2018-319786 30992334

[19] Wagner VM Piver RN Levine MD Backes FJ Chambers LJ Cohn DE. Postoperative venous thromboembolism risk stratification in patients with uterine cancer. Am J Obstet Gynecol. (2023) 228:555.e1–555.e8. 10.1016/j.ajog.2022.12.310 36574873

[20] Wu SS Raymer C Schafer R Culbert A Bernard S Djohan R. Incidence of venous thromboembolism based on Caprini score in deep inferior epigastric perforator flap breast reconstruction. J Reconstr Microsurg. (2023) 39:705–14. 10.1055/a-2040-1532 36809785

[21] Baimas-George MR Ross SW Yang H Matthews BD Nimeri A Reinke CE. Just what the doctor ordered: missed ordering of venous thromboembolism chemoprophylaxis is associated with increased VTE events in high-risk general surgery patients. Ann Surg. (2023) 278:e614–9. 10.1097/SLA.0000000000005779 36538621

[22] Hayssen H Cires-Drouet R Englum B Nguyen P Sahoo S. Mayorga-Carlin M, et al. Systematic review of venous thromboembolism risk categories derived from Caprini score. J Vasc Surg Venous Lymphat Disord. (2022) 10:1401–9. 10.1016/j.jvsv.2022.05.003 35926802 PMC9783939

[23] Olson JD. D-dimer: an overview of hemostasis and fibrinolysis, assays, and clinical applications. Adv Clin Chem. (2015) 69:1–46. 10.1016/bs.acc.2014.12.001 25934358

[24] Sanfilippo KM Fiala MA Feinberg D Tathireddy H Girard T Vij R. D-dimer predicts venous thromboembolism in multiple myeloma: a nested case-control study. Res Pract Thromb Haemost. (2023) 7:102235. 10.1016/j.rpth.2023.102235 38193055 PMC10772878

[25] Hu C Annese VF Barrett MP Cumming DRS. Point-of-care platform for diagnosis of venous thrombosis by simultaneous detection of thrombin generation and D-dimer in human plasma. Anal Chem. (2023) 95:1115–22. 10.1021/acs.analchem.2c03819 36544272 PMC9850404

[26] Yoshikawa T Sano T Terashima M Ikeda M Ioka T Kimura T. Incidence and risk factors for venous thromboembolism in the Cancer-VTE registry stomach cancer subcohort. Gastric Cancer. (2023) 26:493–503. 10.1007/s10120-023-01378-1 37004667 PMC10284943

[27] Chopard R Albertsen IE Piazza G. Diagnosis and treatment of lower extremity venous thromboembolism: a review. JAMA. (2020) 324:1765–76. 10.1001/jama.2020.17272 33141212

[28] Macrae FL Swieringa F Heemskerk JWM Ariëns RAS. High fibrinogen γ' levels in patient plasma increase clot formation at arterial and venous shear. Blood Adv. (2021) 5:3468–77. 10.1182/bloodadvances.2020003346 34438442 PMC8525222

[29] Wolberg AS. Fibrinogen and fibrin: synthesis, structure, and function in health and disease. J Thromb Haemost. (2023) 21:3005–15. 10.1016/j.jtha.2023.08.014 37625698 PMC10592048

[30] Yang Z Rongqing R Yang Z Yang H Yin Y Tian S. Development and validation of a nomogram for predicting deep venous thrombosis in patients with pelvic and acetabular fractures: a retrospective cohort study: Predictive model for pelvic/acetabular fractures. BMC Musculoskelet Disord. (2023) 24:773. 10.1186/s12891-023-06879-9 37784040 PMC10544599

[31] Lv K Yuan Q Fu P Wu G Wu X Du Z. Impact of fibrinogen level on the prognosis of patients with traumatic brain injury: a single-center analysis of 2570 patients. World J Emerg Surg. (2020) 15:54. 10.1186/s13017-020-00332-1 32977824 PMC7517804

[32] Liu Y Deng X Zhu F Zhu W Wang Z. High fibrinogen and mixed proximal and distal thrombosis are associated with the risk of residual venous thrombosis in patients with posttraumatic deep vein thrombosis. Front Cardiovasc Med. (2023) 10:1003197. 10.3389/fcvm.2023.1003197 36818330 PMC9928750

[33] Wang G Zhao W Zhao Z Wang D Wang D Bai R. Leukocyte as an independent predictor of lower-extremity deep venous thrombosis in elderly patients with primary intracerebral hemorrhage. Front Neurol. (2022) 13:899849. 10.3389/fneur.2022.899849 35903126 PMC9314880

